# Supplementary material for: Straightening of light in a one dimensional dilute photonic crystal
Source: Sci Rep. 2019 Oct 1;9:14053. doi: 10.1038/s41598-019-50590-6 (PMC6773682; doi:10.1038/s41598-019-50590-6)
Supplement: Supplementary file 1 — Supplement. Straightening of light in a one dimensional dilute photonic crystal [file 41598_2019_50590_MOESM1_ESM.pdf]

Supplement
Straightening of light in a one dimensional dilute photonic crystal

Zhyrair Gevorkian[1,2*], Vladimir Gasparian[3], and Emilio Cuevas[4]

[1]Yerevan Physics Institute, Alikhanian Brothers St. 2, 0036 Yerevan, Armenia
[2]Institute of Radiophysics and Electronics, Ashtarak-2, 0203, Armenia
[3]California State University, Bakersfield, California 93311-1022, USA
[4]Departamento de Física, Universidad de Murcia, E-30071 Murcia, Spain
[*]gevork@yerphi.am

ABSTRACT

Transmission coefficients for different transverse modes are calculated.

1 Transmission Coefficient

Bloch theorem states that the eigenstate $\phi_{\vec{q}}$ in a periodical potential can be represented in the form

$$\phi_{n\vec{q}}(\vec{\rho}) = e^{i\vec{q}\vec{\rho}} u_{n\vec{q}}(\vec{\rho}), \tag{1}$$

where $u_{n\vec{q}}(\vec{\rho})$ is a periodical function satisfying the equation

$$\left[-\frac{1}{2k_0}(i\vec{q}+\vec{\nabla})^2 + V(\vec{\rho})\right] u_{n\vec{q}}(\vec{\rho}) = E_n(\vec{q}) u_{n\vec{q}}(\vec{\rho}). \tag{2}$$

Below we present the solution of Scrödinger equation Eq.(2) in a unit cell and using it calculate the transmission coefficient. First consider positive energies $E > 0$

$$\begin{aligned} u_{q1}(x) &= (A\cos\beta x + B\sin\beta x)e^{-iqx}, \quad 0 < x < a-b \\ u_{q2}(x) &= (C\cos\alpha x + D\sin\alpha x)e^{-iqx}, \quad a-b < x < a \end{aligned} \tag{3}$$

with $\beta = \sqrt{2k_0 E}$ and $\alpha = \sqrt{2k_0(V_d + E)}$. Remind that $V_d = k_0(\varepsilon - 1)/2$.. Here we omit index n for simplicity.The constants B, C, D can be expressed by A using boundary conditions. A itself can be found from the normalization condition $\int_0^a |u(x)|^2 dx = 1$. Using continuity conditions and periodicity of wave functions one finds dispersion equation in the form

$$\cos(k_0 a \sin\theta) = \cos\alpha b \cos\beta(a-b) - \frac{\alpha^2+\beta^2}{2\alpha\beta}\sin\alpha b \sin\beta(a-b) \tag{4}$$

and following relations between coefficients $C = A$, $D = \beta B/\alpha$ and

$$B = A\frac{\cos\alpha b - e^{ik_0\sin\theta}\cos\beta(a-b)}{e^{ik_0\sin\theta}\sin\beta(a-b) + \frac{\beta}{\alpha}\sin\alpha b} \tag{5}$$

Substituting Eq.(3) into Eq.(15)(main text), using relations between coefficients and taking elementary integrals for a particular contribution of E into transmission coefficient of central diffracted wave one has

$$T = T_1 \times T_2 \tag{6}$$

where

$$T_1 = \frac{1}{a}\left|\frac{\sin\beta(a-b)}{\beta} + \frac{\sin\alpha b}{\alpha} + \frac{\cos\alpha b - e^{ik_0 a sin\theta}\cos\beta(a-b)}{e^{ik_0 a sin\theta}\sin\beta(a-b) + \frac{\beta}{\alpha}\sin\alpha b}\left(\frac{2\sin^2\frac{\beta(a-b)}{2}}{\beta} - \frac{2\beta\sin^2\frac{\alpha b}{2}}{\alpha^2}\right)\right|^2 \tag{7}$$

and

$$\begin{aligned} T_2 = \Bigg[&\frac{a}{2} + \frac{\sin 2\beta(a-b)}{4\beta} + \frac{\sin 2\alpha b}{4\alpha} + \left|\frac{\cos\alpha b - e^{ik_0 a sin\theta}\cos\beta(a-b)}{e^{ik_0 a sin\theta}\sin\beta(a-b) + \frac{\beta}{\alpha}\sin\alpha b}\right|^2 \times \\ &\times\left(\frac{a-b}{2} - \frac{\sin 2\beta(a-b)}{4\beta} - \frac{\beta^2\sin 2\alpha b}{4\alpha^3} + \frac{\beta^2 b}{2\alpha^2}\right) + \\ &+\frac{1}{2}\left(\frac{\cos\alpha b - e^{ik_0 a\sin\theta}\cos\beta(a-b)}{e^{ik_0 a sin\theta}\sin\beta(a-b) + \frac{\beta}{\alpha}\sin\alpha b} + \frac{\cos\alpha b - e^{-ik_0 a sin\theta}\cos\beta(a-b)}{e^{-ik_0 a sin\theta}\sin\beta(a-b) + \frac{\beta}{\alpha}\sin\alpha b}\right) \times \\ &\times\left(\frac{\sin^2\beta(a-b)}{\beta} + \frac{\beta\cos 2\alpha b}{2\alpha^2} - \frac{\beta}{2\alpha^2}\right)\Bigg]^{-1} \end{aligned} \tag{8}$$

Note that Eqs(6-8) determine the contribution of a particular and positive solution of the dispersion equation Eq.(4) ($0 < E < k_0$) into transmission coefficient. In order to find the total transmission coefficient one must, for given parameters a, b, k_0 and ε, find all positive solutions with $E < k_0$ and sum up their contributions. Beside the mentioned positive solutions, one should take into account also the contribution of the negative solutions $E < 0$. The dispersion equation and transmission coefficient for this case can be found from Eqs.(4,7,8) by analytical continuation. The dispersion equation in this case acquires the form

$$\cos(k_0\sin\theta a) = \cos\alpha b\cosh\beta(a-b) - \frac{\alpha^2-\beta^2}{2\alpha\beta}\sin\alpha b\sinh\beta(a-b) \tag{9}$$

where $\beta=\sqrt{2k_0|E|}, \quad \alpha=\sqrt{2k_0(V_d-|E|)}$. Corresponding transmission coefficient has the form

$$T=T_1\times T_2 \tag{10}$$

where

$$T_1=\frac{1}{a}\left|\frac{\sinh\beta(a-b)}{\beta}+\frac{\sin\alpha b}{\alpha}+\frac{\cos\alpha b-e^{ik_0 asin\theta}\cosh\beta(a-b)}{e^{ik_0 asin\theta}\sinh\beta(a-b)+\frac{\beta}{\alpha}\sin\alpha b}\left(\frac{2\sinh^2\frac{\beta(a-b)}{2}}{\beta}-\frac{2\beta\sin^2\frac{\alpha b}{2}}{\alpha^2}\right)\right|^2 \tag{11}$$

and

$$\begin{aligned}T_2=&\left[\frac{a}{2}+\frac{\sinh 2\beta(a-b)}{4\beta}+\frac{\sin 2\alpha b}{4\alpha}-\left|\frac{\cos\alpha b-e^{ik_0 asin\theta}\cosh\beta(a-b)}{e^{ik_0 asin\theta}\sinh\beta(a-b)+\frac{\beta}{\alpha}\sin\alpha b}\right|^2\times\right.\\&\times\left(\frac{a-b}{2}-\frac{\sinh 2\beta(a-b)}{4\beta}+\frac{\beta^2\sin 2\alpha b}{4\alpha^3}-\frac{\beta^2 b}{2\alpha^2}\right)+\\&+\frac{1}{2}\left(\frac{\cos\alpha b-e^{ik_0 a\sin\theta}\cosh\beta(a-b)}{e^{ik_0 asin\theta}\sinh\beta(a-b)+\frac{\beta}{\alpha}\sin\alpha b}+\frac{\cos\alpha b-e^{-ik_0 asin\theta}\cosh\beta(a-b)}{e^{-ik_0 asin\theta}\sinh\beta(a-b)+\frac{\beta}{\alpha}\sin\alpha b}\right)\times\\&\left.\times\left(\frac{\sinh^2\beta(a-b)}{\beta}+\frac{\beta\cos 2\alpha b}{2\alpha^2}-\frac{\beta}{2\alpha^2}\right)\right]^{-1}\end{aligned} \tag{12}$$

Now using the above mentioned expressions one can calculate transmission coefficient of central diffracted wave. Taking $a=0.6\mu m, \quad b=0.06\mu m, \quad \varepsilon=4 \quad k_0=12\mu m^{-1}$ and numerically calculating we get for $\theta=0$, $E_s=2.227$, $T_s=0.358$, $E_m=4.436$, $T_m=0$ $|E_b|=4.729$,$T_b=0.626$. Here $E_s,E_m<12$ are the positive solution of dispersion equation Eq.(4), $T_{s,m}$ are the corresponding partial transmission coefficients calculated using Eq.(8). Correspondingly E_b,T_b are contributions from negative solution calculated using Eqs.(9) and (12). The resulting transmission coefficient for incident angle $\theta=0$ is $T=T_s+T_b+T_m=0.984$. For any other incident angle the transmission coefficient can be calculated in analogous manner. We present results also for incident angle $\theta=\pi/12$, $E_s=1.403$,$T_s=0.639$, $\quad E_m=6.259$,$T_m=0.008$ $\quad |E_b|=4.686$,$T_b=0.258$ $T=T_s+T_b+T_m=0.905$. For $\theta=\pi/4$, $E_s=1.748$, $T_s=0.496$, $\quad E_m=5.331$, $T_m=0.002$, $\quad |E_b|=4.708$, $T_b=0.448$ $T=T_s+T_m+T_b=0.946$. As it is obvious from these calculations the main contribution to the transmission coefficient of central diffracted wave give s,b modes. The contribution of the m mode with not small positive energy is negligible and not shown in band scheme Fig.3.The angle dependence of transmission coefficient is presented in Fig.2 of main text. Note that in the vacuum case $b=0$ there is no localized mode and positive energy as it follows from Eq.(4) equal $E_m=k_0\sin^2\theta/2$. We use this value when calculating transmission coefficient in vacuum case, see Fig.2.(main text)
